# Surfactant-enabled strategy for molecular solar thermal energy storage systems in water

**DOI:** 10.1039/d5gc04357c

**Published:** 2025-10-15

**Authors:** Lorette Fernandez, Helen Hölzel, Pedro Ferreira, Nicolò Baggi, Kévin Moreno, Zhihang Wang, Kasper Moth-Poulsen

**Affiliations:** a Department of Chemical Engineering, Universitat Politècnica de Catalunya, EEBE Eduard Maristany 10-14 08019 Barcelona Spain helen.holzel@upc.edu kasper.moth-poulsen@upc.edu; b Institute of Organic Chemistry, Justus-Liebig-University Giessen Heinrich-Buff-Ring 17 35392 Giessen Germany; c School of Engineering, College of Science and Engineering, University of Derby Markeaton Street Derby DE22 3AW UK; d Department of Materials Science and Metallurgy, University of Cambridge Cambridge CB3 0FS UK; e Department of Chemistry and Chemical Engineering, Chalmers University of Technology 41296 Gothenburg Sweden; f The Institute of Materials Science of Barcelona, ICMAB-CSIC Bellaterra 08193 Barcelona Spain; g Catalan Institution for Research & Advanced Studies, ICREA Pg. Lluìs Companys 23 08010 Barcelona Spain

## Abstract

Molecular solar thermal energy storage (MOST) systems, which absorb sunlight, store this energy in chemical bonds, and release it as heat, are receiving increasing attention in renewable energy storage applications. Among the norbornadiene/quadricyclane (NBD/QC) couples developed for MOST, the 2,3-difunctionalized cyano- and *p*-aryl-substituted NBD/QC couples have received greater attention for their promising properties. However, their application in solution requires the use of hazardous solvents, which limits their potential for large-scale implementation. Here, new greener systems consisting of cyano- and *p*-alkoxyphenyl-substituted NBD/QC derivatives dissolved in non-ionic surfactants and water were investigated. Concentrations of NBD up to 1.6 M were achieved by tuning the water/surfactant ratio, meeting the solubilization properties of organic solvents. The most promising system was further characterized, and its properties in water-based solutions were compared with those observed in toluene. Integration into a solar energy-harvesting liquid device led to the full conversion of the NBD to QC. The evaluation of the heat release performance upon catalytic trigger resulted in a temperature increase of 4.7 °C in ambient conditions. This demonstrates that promising NBDs/QCs can be used for MOST in aqueous media without compromising key performance parameters such as energy density, photoconversion, and catalyzed heat release.

## Abstract 2

A surfactant-water-based approach is applied as a safer and more environmentally friendly alternative to toluene for molecular solar thermal energy storage using norbornadiene-quadricyclane derivatives.

## Graphical abstract

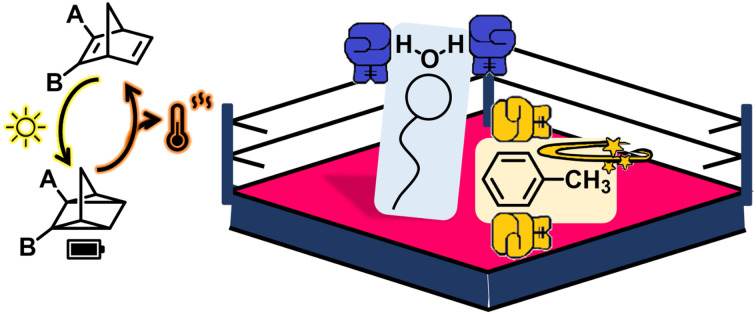

**Green foundation**

1. This work presents a sustainable alternative to the highly toxic and flammable solvent toluene, which is commonly used to dissolve norbornadiene (NBD) derivatives in molecular solar thermal energy storage (MOST) applications. A surfactant/water-based system is introduced, enabling the solubilization of NBD at concentrations up to 1.6 M, with performance comparable to traditional organic media.

2. The green aqueous system leads to a marked improvement in MOST systems’ performance: a 30% increase in photoisomerization quantum yield and a thermal energy storage duration more than three times longer than that of toluene-based systems. These enhancements demonstrate the feasibility of a more efficient and environmentally friendly MOST system.

3. The practical implementation of the system is validated through full NBD-to-quadricyclane (QC) conversion under simulated sunlight and on-demand energy release *via* catalytic back-conversion. A temperature increase of 4.7 °C under ambient conditions confirms the ability of this water-based system to function as both a solar energy storage medium and a reliable system for heat release.

## Introduction

With the growing awareness of carbon emissions and diminishing fossil fuel reserves, the world is facing a steadily increasing demand for sustainable energy. Among renewable sources, solar energy stands out due to its abundance and widespread availability. Over the years, several methods have been developed and explored to capture, convert, and store solar energy, as well as to address the challenges posed by its daily and seasonal availability and local intermittency. Examples include photovoltaic panels combined with batteries^1,2^ or hydrogen production by water splitting.^3,4^ A promising emerging approach is molecular solar thermal energy storage (MOST) systems, which store solar energy within the chemical bonds of high-energy metastable photoisomers and release it as heat or, through thermoelectric conversion, as electricity, on demand.^5–8^ Several types of photoswitchable molecular systems have been identified, such as anthracenes,^9–11^ diarylethenes,^12^*trans*/*cis*-azobenzene,^13–15^ dihydroazulene/vinylheptafulvene,^16^ or norbornadiene/quadricyclane (NBD/QC).^17–19^ An ideal MOST system needs to fulfill specific requirements, as recently reviewed by Wang *et al.*^5^ Key criteria for these systems include a molecular absorption range that closely matches the solar spectrum, a photoisomerization quantum yield (*Φ*_iso_) near 100%, a long thermal half-life (*t*_1/2_) at room temperature (ranging from days to years), high energy storage density (Δ*H*_storage_) – at least 0.3 MJ kg^−1^ to surpass conventional heat storage materials, an efficient triggering mechanism for energy release, the ability to undergo numerous conversion and back-conversion cycles without degradation, distinct absorption spectra for each isomer to prevent optical overlap.^5^ Moreover, for practical application, benign environmental impact, non-toxicity, and non-flammability are of paramount importance. Although developing a MOST system meeting all these criteria remains a challenge, certain compromises are necessary, and ongoing research continues to optimize these systems.

Major improvements have been achieved in the molecular design of NBD/QC derivatives to meet the aforementioned criteria.^18,20–24^ The NBDs conversion is driven by a photoinduced [2 + 2] cycloaddition to their valence isomer, QC.^25^ The back-conversion can be triggered thermally,^26,27^ electrochemically,^28^ optically,^29^ or catalytically.^30–32^ Recently, the 2,3-difunctionalized cyano- and *p*-methoxyphenyl-substituted NBD (NBD1) has received greater attention due to its cycling robustness, good ambient stability (*t*_1/2_ = 30 days at 25 °C) and high solubility in toluene (1.52 M), which led to a record thermal gradient, under vacuum conditions in a flow system (Δ*T* = 63 °C). In addition to its low molecular weight, the molecule also exhibits a good solar spectral match and high energy density (0.4 MJ kg^−1^).^24,33,34^ Moreover, this NBD can be produced on a large scale using continuous flow chemistry.^35,36^

When operating such MOST systems in solution, solubility is a key parameter: the higher the concentration of the photoswitch, the higher the possible temperature gradient upon heat release.^5,24,37^ As mentioned above, toluene displays good solubilization properties and it is often the preferred solvent, as it does not interfere with the NBDs’ absorbances (UV cutoff at 284 nm). This solvent also has a relatively high boiling point (110 °C) and a low specific heat capacity of 1.7 J g^−1^ °C^−1^, which is beneficial during the heat release process.^24^ However, its toxicity and flammability could limit future large-scale implementation. Indeed, even if the combination of its environmental, health, and safety properties (EHS, lower than 3.5) and its life-cycle assessment (LCA, around 20) makes toluene favorable, compared with other solvents, Tobiszewski *et al.* ranked it 59^th^ out of 78 solvents, not indicative of low environmental risk.^38,39^ A significant step forward in the application of relevant NBD/QC derivatives in solution would be the use of water as solvent.^40^ The water solubility of various photoswitches has already been the focus of several studies, especially in the field of photopharmacology.^41^ Investigations regarding NBD in aqueous media were reported employing octa acid^42^ to encapsulate the unsubstituted NBD or using an alkaline medium where carboxylic groups are deprotonated.^44,45^ Recently, NBD was chemically modified by introducing highly water-soluble groups (amines). This derivative achieved macroscopic heat release at optimized temperatures, using gold nanoparticles as a catalyst trigger.^43^

**Fig. 1:**
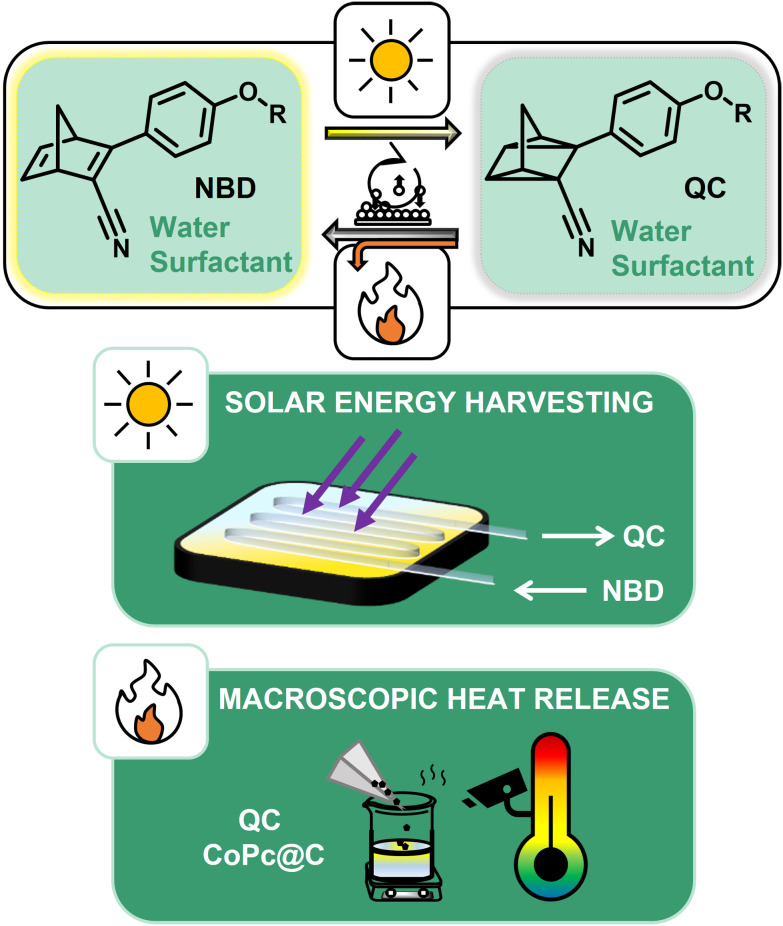
Water-compatible systems involving NBD/QC derivatives (R = CH_3_, C_8_H_17_) and non-ionic surfactants. MOST principle: molecular solar energy storage and heat release upon catalytic trigger.

**Fig. 2:**
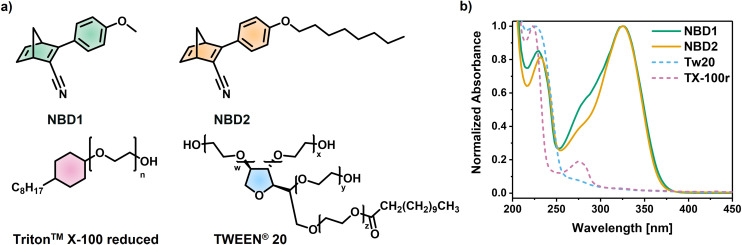
(a) Structures of the photoswitches (NBD1 and NBD2) and the non-ionic surfactants (Tw20 and TX-100r), used in this work. (b) Normalized absorbance spectra (water baseline) of NBD1 and NBD2 in water/TX-100r, Tw20, and TX-100r in water.

In this work, to further explore the aqueous alternative and better match some of the MOST systems’ requirements, new water-compatible systems involving NBD/QC derivative and non-ionic surfactants are presented (Fig. 1). A suitable surfactant was identified for the aqueous mixtures with NBDs having lower and higher hydrophobicity. The methoxy group of NBD1 was replaced with an octyloxy substituent (NBD2) to show that this approach can be applied to even more hydrophobic NBDs. Their structures are displayed in Fig. 2a, and their photophysical properties in water-based solutions were evaluated. The most promising system was integrated into a liquid device, assessed with regard to its catalyzed heat release potential, and compared with a toluene-based approach. This study introduces a novel MOST design strategy that replaces harmful organic solvents with water and non-ionic surfactants, offering a sustainable pathway for the future development of environmentally friendly MOST systems.

## Results and discussion

While NBD1 has already been extensively characterized in toluene,^24,33,34^ its behavior in water-based systems remains unexplored. Herein, the potential use of an alternative medium involving non-ionic surfactants is further investigated, as is the tuning of the photoswitch hydrophobicity.

### Determination of a suitable surfactant

Surfactants are amphiphilic molecules that lower the tension of the surface on which they are adsorbed due to repulsive electrostatic or steric interactions. At high interfacial tension between water and their hydrophobic regions, they self-assemble by pulling the hydrophobic moieties away from the aqueous phase and bringing the hydrophilic moieties into contact with it. As a result, they induce organic hydrophobic areas in water that can entrap hydrophobic compounds, enabling their dispersion in an aqueous environment.^46–49^ Ionic surfactants, which feature small and strongly hydrophilic charged head groups, prevent the organic phase aggregation by electrostatic repulsive forces. On the other hand, non-ionic surfactants, which differ in having long and weakly hydrophilic head groups, reduce the aggregation by hydration, thermal fluctuations, and steric hindrance. While ionic surfactants might cause irritation at high concentrations, non-ionic surfactants are biocompatible, less toxic, and more stable.^48–50^ Since this work aims to propose a greener alternative to toxic organic solvents, and high surfactant contents are expected to be involved in a potential application, the non-ionic option was selected. Depending on the surfactant content, the amount of scattered light, *i.e.*, turbidity, cloudiness, or opacity, and thus the clarity of a mixture might be affected.^51,52^

In this work, clear dispersions were created to prevent potential filtering effects or optical scattering due to high concentration-induced opacity, and to ensure a direct comparison with the NBDs in toluene.††It should be mentioned that the cloud point temperature (65 °C and 76 °C, for TX-100r and Tw20, respectively), above which water molecules would no longer interact with the surfactant, forming a distinct phase, is not relevant in the study. This threshold is usually determined at 1–2% of surfactant in solution and is considerably higher with increasing concentration^71^ while here, the surfactant content ranges from 28 to 84% in water.

First, two commercially available surfactants (TWEEN® 20, named as Tw20, and Triton™ X-100 reduced, named as TX-100r, in Fig. 2a were selected to prepare the aqueous system. Tw20 is one of the most commonly used non-ionic surfactants. It finds applications in cosmetics, food (being approved as a food additive in the European Union), and biopharmaceutical industries.^46,47,53–56^ In 2018, 40% of liquid protein formulations involved Tw20.^57^ With a long polyoxyethylene chain and a fatty acid ester group (lauric acid), this polysorbate is water-soluble and displays a wide range of hydrophobicity and surface activity, which are beneficial for stabilizing oil/water emulsions.^46,53^ Alternatively, TX-100r is the hydrogenated version of Triton™ X-100. The latter was also widely used in the biopharmaceutical sector for protein-membrane solubilization or virus inactivation. However, its utilization was restricted by the European Chemicals Agency due to its degradation products that may be harmful to the environment.^46,58,59^ As an eco-friendly alternative, TX-100r, made of polyethylene glycol (PEG) and alkylcyclohexyl moieties, is structurally comparable to Triton™ X-100, with a fully hydrogenated six-membered carbon ring. Both display similar properties, while TX-100r is not classified as bioaccumulative or toxic.^59,60^ Another important feature of Tw20 and TX-100r is the lack of overlap between their absorbance spectra and that of the studied NBDs, as shown in Fig. 2b.^24,46,60^ Besides, they showed no photodegradation under irradiation at 340 nm for 10 min (Fig. S1).

NBD2 was identified as a viable alternative to NBD1 to assess the versatility of the concept in accommodating variations in photoswitch hydrophobicity. Indeed, NBDs do not require any specific hydrophilic groups or acidic/basic pH conditions to be processed in the surfactant/water mixtures studied here. Thus, the present approach opens the path to a collection of NBDs that would generally not be used in aqueous medium. Typically, the aqueous solubility of organic materials is shown to be worse with longer chains.^61,62^ Therefore, in this work, NBD's hydrophobicity is tuned by only changing the length of the alkoxy chain to preserve the push–pull effect and, hence, the good optical properties.

**Fig. 3:**
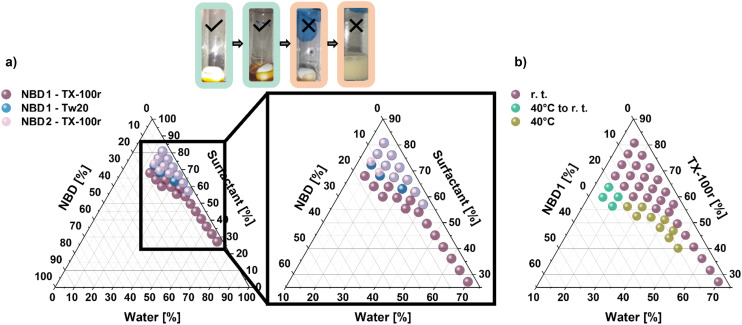
Study of the mixing of NBDs, non-ionic surfactants, and water. Light green bordered photos: clear, homogenous mixtures suitable for the investigation; light orange bordered photos: heterogeneous mixtures. Ternary diagrams: (a) NBD1 with TX-100r and Tw20, and NBD2 with TX-100r; (b) temperature influence on the mixtures involving NBD1, TX-100r, and water (*i.e.*, at room temperature, preparing the mixture at 40 °C and allowing it to cool to room temperature, and preparing it and maintaining it at 40 °C).

In order to map out the structure–property relations of the employed molecules and surfactants, ternary diagrams in water were plotted. Due to the high concentrations of NBDs and surfactants and to avoid saturation of the UV-Vis detector (Tables S1, S2, and S3), only naked-eye assessment was used to estimate the level of turbidity of the mixture, rather than the conventional method using UV-visible spectroscopy.^51^ Initial NBD/surfactant mixtures (5/95 to 30/70 wt%) were prepared at room temperature by stirring NBD and surfactant together for 5 min. Then, water was added gradually (5% by 5%) to the multiple NBD/surfactant mixtures. Each sample was stirred for at least 30 min at an appropriate temperature, as detailed below. The homogeneous transparent solutions obtained with different ratios of NBD, surfactant (TX-100r or Tw20), and distilled water are depicted in Fig. 3. This study aimed to obtain clear solutions while reaching the highest NBD and water contents with a minimum amount of surfactant. The lowest contents of NBDs and water were set at 3% and 15% respectively, to ensure a certain minimum operational load in the final mixture.

**Fig. 4:**
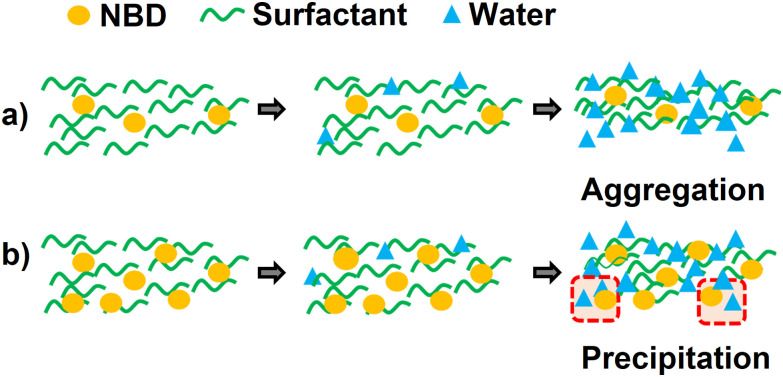
Schematic of (a) aggregation and (b) precipitation processes.

The transparency loss at given ratios can be attributed to the formation of aggregates. At high surfactant concentrations (0.6 to 8.6 M), the photoswitch and water are likely dispersed within the surfactant-rich matrix. However, as water is gradually added and surpasses a critical threshold, the system undergoes a phase inversion in which water becomes the continuous phase. This shift forces the surfactant molecules to reorganize, potentially leading to aggregate formation, as illustrated in Fig. 4a.

First, TX-100r and Tw20 were compared at room temperature using NBD1. TX-100r (dark violet) offers a wider working range than Tw20 (blue), depicted in Fig. 3a. While the NBD maximum contents fall within the same range (17% with TX-100r – *i.e.*, 0.9 M, 13% with Tw20 – *i.e.*, 0.7 M), higher water ratios were reached with the TX-100r. Indeed, the latter can achieve 70% water content, compared with 40% with Tw20. A possible explanation could be the formation of larger aggregates for identical mixtures, due to the longer alkyl chain length of Tw20, compared to TX-100r, which leads to more light scattering.^63^ It should be noted that at higher contents of NBD1, a precipitate is formed during the addition of water. This is attributed to the saturation of the surfactant medium. Similar events were observed in drug encapsulation involving a non-ionic surfactant.^64^ The organic compound likely leaches from the surfactant medium to the aqueous phase, resulting in a mixture that is no longer homogeneous, as depicted in Fig. 4b.

Since TX-100r was the most suitable surfactant for NBD1, it was then tested at room temperature with NBD2. The working range of NBD2 (light violet), as displayed in Fig. 3a, is smaller. The maximum of NBD content is in the same order (12% – *i.e.*, 0.6 M) as for NBD1, but only up to 40% water is achieved. As expected, due to its longer chain that promotes aggregation, larger agglomerates were formed in the presence of NBD2 for contents of surfactant and water that were still leading to clear homogenous solutions with NBD1. Therefore, the accessible ratios of NBD and water are lower with this photoswitch. Nevertheless, concentrations of 0.14 M with the mixture containing 40% water, or even 0.6 M with a further halving of the water content, are achieved, which is still reasonable for integration into solar harvesting devices (Table S3).

Since the most promising concentrations could be achieved with NBD1 and TX-100r, the influence of a higher temperature of preparation was explored to extend the concentration range, as shown in Fig. 3b. When the mixtures initially turned opaque after the water addition, they were heated to 40 °C and allowed to cool back to room temperature. If still transparent, water was added again, and the same operation was repeated. This allowed the concentration of NBD1 to be raised to 1.6 M, meeting the solubilization properties of toluene (Table S1). When the mixtures were becoming opaque while cooling, the temperature was increased and maintained at 40 °C. An extended range of transparency was observed, as depicted in Fig. 3b. The water content increases with heating can be exemplified with the mixture involving a water content of 25% at ambient temperature that is doubled to 50% at 40 °C, while still ensuring a reasonable amount of NBD1 (from 15% to 10%) (Table S1). A similar statement can be made from an NBD standpoint. For instance, in a mixture involving 50% water, heating allows the content of NBD1 to be doubled compared to ambient temperature (10% *vs.* 5%).

These observations are likely due to the disruption of hydrogen bonds in the water molecules’ network at higher temperatures, which offers more mobility to the NBD/surfactant and prevents aggregation.^65^ Thus, an approach to improve the NBDs content in a water-based solution could be to work at higher temperatures. The functioning temperature of a MOST fluid can be varied massively depending on the irradiation conditions. As a proof of concept, to test the system performance under fixed, unchanging conditions, and to ensure comparability with the solutions reported in toluene, given that they were prepared at room temperature, the next steps of the study were performed at room temperature.

### Photophysical properties of NBDs in water-surfactant *vs.* toluene

The properties of the NBDs in the water-based system for MOST applications were investigated.

Their molar extinction coefficients, displayed in Table 1, are determined to be of the same order of magnitude (Fig. S3).

**Table 1:** Photophysical properties of NBDs, at room temperature, reported in toluene^24,66^ and those determined in water/TX-100r: wavelengths of absorbance maxima (*λ*_max_) and absorbance onsets (*λ*_onset_), and molar extinction coefficient at the wavelengths of absorbance maxima (*ε*_max_)

		*λ*_max_ (nm)	*λ*_onset_ (nm)	*ε*_max_ (M^−1^ cm^−1^)
NBD1	Toluene	326	380	13 300 ± 203
	Water/TX-100r	326	380	11 800 ± 560
NBD2	Toluene	328	397	16 600 ± 101
	Water/TX-100r	326	385	13 200 ± 402

**Fig. 5:**
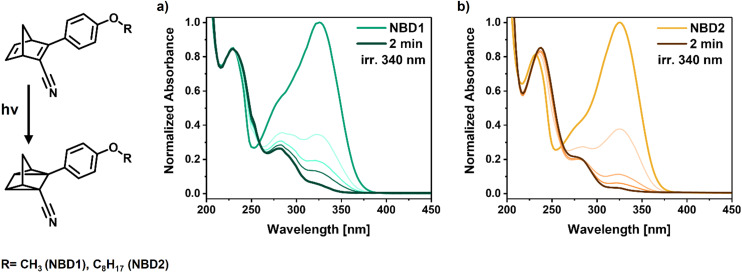
Normalized absorbance spectra of the photo-conversion of the photoswitches to the respective QC derivative (a) NBD1 and (b) NBD2 in the water-TX-100r-based system.

The conversion of the NBDs was examined by UV-vis spectroscopy. An initial study with NBD1 highlights that, even in the absence of aggregation, compared to toluene, a portion of NBD1 may remain undissolved if the ratio of NBD/surfactant is higher than 2/98 (Fig. S4). Thus, for NBD1, a higher surfactant content was used to ensure full dissolution. In the case of NBD2, while a portion may also remain undissolved, the photoswitch aggregates up to 2/98 of NBD/surfactant (Fig. S5). Indeed, compared to NBD1, the alkyl chain of NBD2 offers more mobility into the hydrophobic area. Since in diluted media, the NBDs and TX-100r are likely forming suspended particles, this mobility induces the expansion of the particles’ volume in the NBD2 system, which, as expected, scatters light. By increasing the surfactant content, the aggregation fades. Nevertheless, conversion from NBD to QC is successfully achieved with both NBDs as suggested in Fig. 5 by the progressive decrease of the bands at 326 nm with isosbestic points at 259 and 261 nm for NBD1 and 2, respectively, reflecting clean NBD to QC isomerization.

These observations make the new water-based approach promising as a medium for NBDs, since it overcomes the challenge of dissolving the hydrophobic molecules in water. As the combination of NBD1 and TX-100r displays an extended transparency working range, *i.e.*, higher achievable NBD contents, it was exposed to more in-depth studies. The conversion in the water-based medium was confirmed by ^1^H-NMR spectroscopy while performing a test at a higher NBD1 concentration (0.02 M *vs.* ≈ 10^−5^ M in the UV-vis spectroscopy study) to achieve 96% of QC1 (Fig. S6). In this experiment, a highly concentrated solution of surfactant (1.6 × 10^−1^ M *vs.* 10^−4^ to 5 × 10^−3^ M in the UV-vis spectroscopy study) is involved. This underlines the versatility of the approach regarding the NBD and surfactant contents. Indeed, independently of the concentrations of NBD1 and TX-100r in water, the conversion occurs. It is worth noting that NBD1 could be isolated from the surfactant and recovered by extraction followed by column chromatography (Fig. S14).

From this point on, the work focuses on the behavior of NBD1/QC1 in the water-based medium compared with toluene, and the data are displayed in Table 2. While the NBD1 molar extinction coefficient is also of the same order of magnitude as in toluene, its quantum yield is improved by 30% in the water-based system (Table S4). Based on reported solvent effects, this outcome is unexpected.^67^ Indeed, the present solvent system is mainly made of water (the most polar solvent). However, herein, it is hypothesized that the NBD does not directly interact with the aqueous medium but is surrounded by the surfactant. Sabirov *et al.* reported the enhancement of the thermodynamic favorability of NBD conversion while decreasing the size of fullerene cages into which the photoswitch was encapsulated.^68^ In this way, the improved quantum yield in the present study could be possibly attributed to the local confinement of NBD1 induced by the surfactant, which might favor the photoconversion process.

**Table 2:** Photophysical properties of NBD1, at room temperature, reported in toluene^24^ and those determined in water/TX-100r: isomerization quantum yield (QY), half-life (*t*_1/2_), enthalpy (Δ*H*^‡^_therm_), entropy (Δ*S*^‡^_therm_), Gibbs free energy (Δ*G*^‡^_therm_) of back-reaction

	QY (%)	*t*_1/2_ at 25 °C (days)	Δ*H*^‡^_therm_ (kJ mol^−1^)	Δ*S*^‡^_therm_ (J K^−1^ mol^−1^)	Δ*G*^‡^_therm_ (kJ mol^−1^)
Toluene	61	30	104	−22	111
Water/TX-100r	79	112	117	11	114

**Fig. 6:**
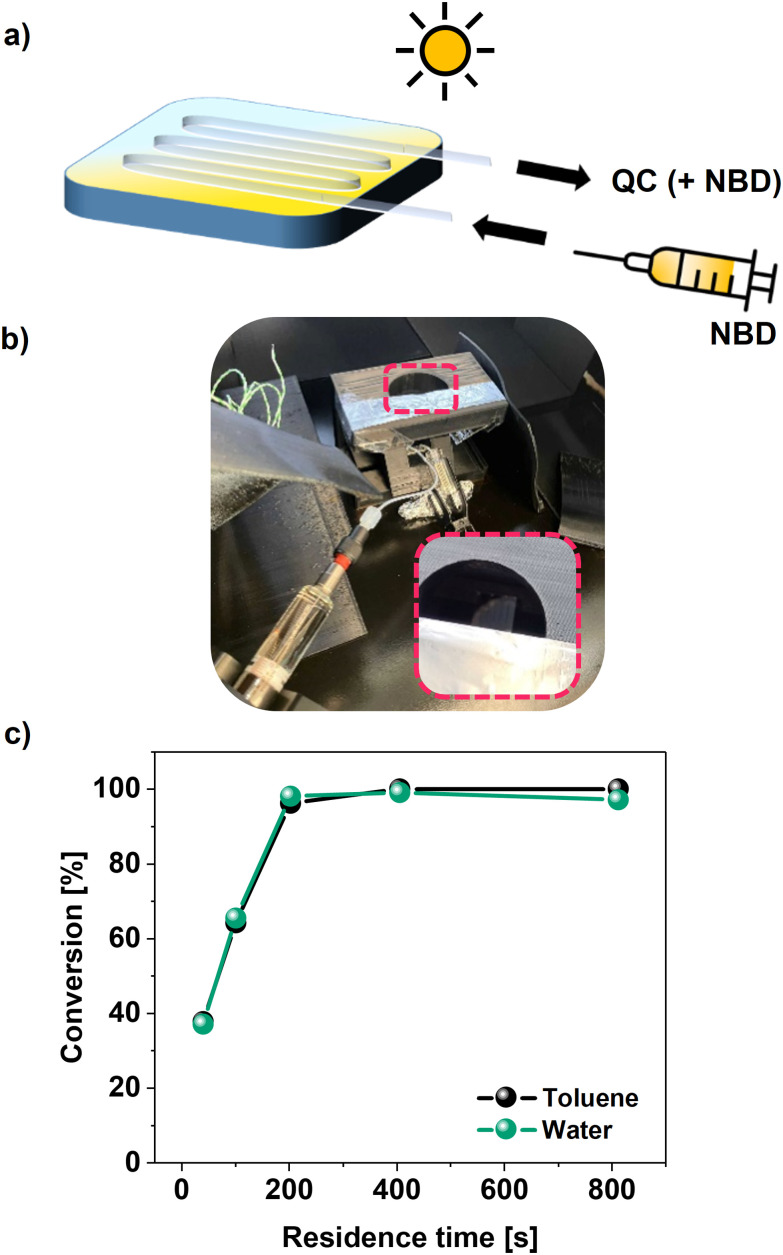
Integration into a solar energy harvesting liquid device. (a) Schematic of the device, (b) picture of the setup, (c) comparison of the conversion percentages from NBD1 (0.14 M) to QC1 with the increased residence times in the chip, between systems involving TX-100r with toluene or water.

To further investigate the conversion in this greener medium under more application-relevant conditions, its integration into a small solar harvesting liquid device was demonstrated with the NBD1/TX-100r mixture with 69% water content. Solutions of NBD1 (0.14 M) with TX-100r (28%) were pumped at different flow rates through a fused silica microfluidic chip, under simulated sunlight irradiation, as illustrated in Fig. 6a and b. To simulate sunlight conditions, a solar simulator, calibrated to the corresponding irradiation of 1 sun, was used. The conversion of NBD1 to QC1, as well as the TX-100r stability, were evaluated for the different residence times (Table S5 and Fig. S2, S9). As shown in Fig. 6c, both systems involving NBD1 in TX-100r and toluene or water display the same trend in conversion percentages for the corresponding residence times. Considering the integration error within the 5% with standard parameters used in ^1^H-NMR, these results are in line with those reported for NBD1 (0.1 M) in pure toluene, where 97% conversion is achieved with extended residence time under solar irradiation.^69^

Having demonstrated that NBD1 in a TX-100r/water system can easily meet the conversion properties of NBD1 in toluene, the back-conversion process was investigated.

Thermal and catalytic back-conversions of QC1 were evaluated in the water-based medium. An initial assessment was performed at a low concentration with negligible surfactant content (*c*_TX-100r_ ≈ 10^−5^ M) compared to water. The thermal back-conversion was achieved while performing a kinetics study at different temperatures (Fig. S7 and S8). The data extracted from the reaction rates and already reported in toluene are displayed in Table 2.^24^ While the enthalpy of back-reaction (Δ*H*^‡^_therm_) is of the same order of magnitude as that reported in toluene, the positive Δ*S*^‡^_therm_ indicates an increase in the disorder. This is likely due to the local confinement induced by the surfactant. Herein, a spontaneous reaction should be favored, unlike in toluene. However, the difference in the Gibbs free energy is only 3.3 kJ mol^−1^, while the half-life of QC1*t*_1/2_ is more than 3.7 times longer than in toluene. Perhaps the surfactant induces additional structural changes at the transition state (TS), which imply more energy is required to reach the TS. Further investigation would be beneficial in understanding the processes involved, but it is beyond the scope of this study. Regardless, these results align with the solvent polarity trend reported for various QCs exhibiting extended storage times in more polar solvents.^19,66^ This thermal pathway was also studied at a higher concentration (*c*_NBD1_ ≈ 0.02 M) by ^1^H-NMR, where QC1 was fully converted back to NBD1 after heating the sample at 80 °C overnight (Fig. S6).

**Fig. 7:**
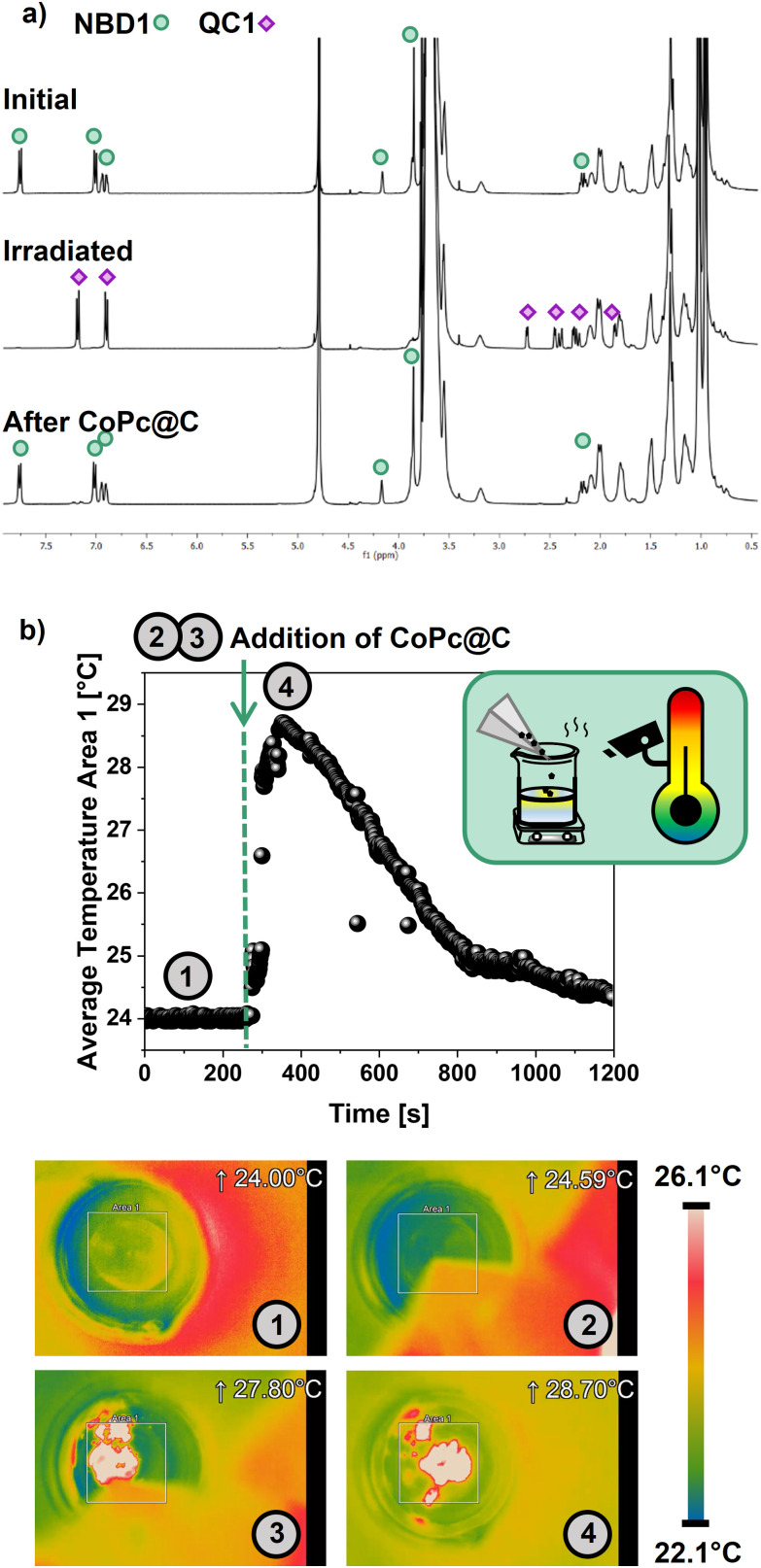
Catalytic back-conversion. (a) ^1^H-NMR spectra of the converted NBD1 to QC1 and after 3 h following the addition of CoPc@C, (b) evolution of the average temperature of area 1 (values in white on the corresponding images) function of time and corresponding images of the heat release experiment monitored by thermal camera from 1 (initial, QC1 0.6 M in water/TX-100r), 2 (before adding the catalyst), 3 (during) to 4 (after the addition), and evolution of the temperature function of time in the area 1. The reference scale bar refers to 4 and displays the hottest and the coldest temperatures of the full image.

From a practical perspective, catalytic triggers are the preferred choice for rapid heat generation in solution. The record heat release for QC1 had been reported in toluene with cobalt phthalocyanine physisorbed on activated carbon support (CoPc@C).^24^ For the sake of comparison, this catalyst was tested in the water-based system. The full back-conversion to NBD1 was confirmed after 3 hours by ^1^H-NMR (Fig. 7a), proving that a catalytic back-conversion could also be achieved in this medium.

In the context of a MOST application, the on-demand macroscopic heat release was also demonstrated as a proof-of-concept. The study was performed in ambient conditions with converted QC1 (0.6 M) solutions in toluene as a reference experiment, and in water/TX-100r. This concentration was selected to ensure a greater proportion of QC1 than water, which is viewed as part of the solvent. Indeed, according to the ternary diagram, a higher concentration could have been considered to maximize the heat release,^24^ but this would have implied a lower water content, which is not within the scope of this work. CoPc@C, with a 5% weight ratio of catalyst to QC1, was added in a single shot into the solution under stirring while recording the average temperature with a thermal camera (Fig. S10). As displayed in Fig. 7b, as soon as the catalyst is in contact with the QC1 solution, the temperature rises instantaneously, which reflects the back-conversion to NBD1, confirmed by ^1^H-NMR (Fig. S13 and Supplementary Movie S1). The experiment led to a macroscopic heat release Δ*T* of at least 4.7 °C in the aqueous medium. Indeed, it is worth noting that the temperatures displayed in Fig. 7b are the average temperatures of area 1 and not temperature peaks of the area, which means that the actual Δ*T* is higher. In the control experiment in toluene, the back-conversion to NBD1 (Fig. S11, S12, and Supplementary Movie S2) achieved a Δ*T* of 14.6 °C. This greater Δ*T* was expected due to the lower specific heat capacity of toluene (1.7 J g^−1^ °C^−1^*vs.* 4.18 J g^−1^ °C^−1^ for water).^24^ Such an outcome is also supported by the theoretical heat release values that were predicted by using eqn (1) (Methods). Theoretical Δ*T* were estimated in pure toluene, water, and TX-100r with a QC1 concentration of 0.6 M. Theoretical heat capacity of QC1 and the aforementioned heat capacities of toluene and water were used. Since the specific heat capacity of TX-100r was unknown, it was determined to be 3.34 J g^−1^ °C^−1^ by differential scanning calorimetry (Fig. S15). The theoretical Δ*T* are 23.7 °C, 10.7 °C, and 12.6 °C in pure toluene, water, and TX-100r, respectively, not considering heat losses. The theoretical Δ*T* in a water/TX-100r solution can therefore be expected to be between 10.7 °C and 12.6 °C. Considering the reference experiment in toluene, the difference between experimental and theoretical results is comparable for water/TX-100r, *i.e.*, 9.1 °C *versus* 6.0 °C to 7.9 °C, which highlights the promising proof of principle of the water/surfactant-based system. Indeed, if heat losses are reduced and these temperatures are achieved, taking into account that the technology used here to measure heat release can be adjusted to be more accurate, *i.e.*, monitoring a smaller area, this heat could be sufficient to increase the ambient temperature by about 10 °C.

The overall study demonstrated that the water-compatible approach with NBDs is promising for MOST applications. The properties of the NBD1 in the water-based system for MOST application are comparable to those reported in toluene or even better, to some extent. The integration into a solar harvesting liquid device was successful, as evidenced by a macroscopic heat release recorded in an aqueous system.

## Conclusions

This work reveals the great potential of using a non-ionic surfactant for an efficient MOST water-compatible system based on cyano- and *p*-alkoxyphenyl-substituted NBD/QC. Triton™ X-100 reduced provides an extended working range. Mixtures involving NBD1 are the most promising. Given an NBD concentration of 0.14 M, 70% water is achieved with NBD1, *versus* 40% with NBD2, due to the long chain that promotes the aggregation. A further mixture resulted in 1.6 M of NBD1 while increasing the temperature. This NBD concentration meets the solubilization properties of the toluene. The photophysical characterization of the new system highlighted a 30% improvement in the photoisomerization quantum yield and a three-times longer storage time than in toluene. The system was successfully integrated into a solar energy harvesting liquid device, and a macroscopic heat release of 4.7 °C was determined upon catalytic trigger in ambient conditions. This new study opens opportunities for a more sustainable and greener implementation of NBD/QC couples in large-scale MOST devices. Future water/surfactant-based solvents designed for MOST application should focus on cheaper surfactants, to reduce the final cost of the device, that display lower heat capacities for better heat transfers.

## Experimental

Triton™ X-100 reduced and TWEEN® 20 were purchased from Sigma-Aldrich and used without further purification.

Distilled water was used throughout the study.

Toluene used for the flow conversion was HPLC grade, purchased from VWR, and used without further purification.

Toluene-*d*_8_, deuterium oxide (D_2_O), and chloroform-*d* (CDCl_3_) stabilized with silver foils were purchased from Eurisotop, and used without further purification.

The CoPc@C was prepared following a reported procedure.^24^

### Synthesis

3-(4-Methoxyphenyl)-2-propynenitrile was prepared according to a literature procedure in flow.^36^NBD1 was synthesized *via* combined cracking and Diels–Alder reaction from the acetylene in the presence of dicyclopentadiene, following the published flow synthesis procedure.^35^

NBD2 was synthesized according to a synthetic procedure to be published elsewhere.^66^

### Ternary diagrams

Mixtures with different ratios of NBD/surfactant/water were prepared as follows. The surfactant was placed into a 3 mL vial, followed by the addition of NBD, and stirred for 5 min to obtain a clear yellow homogeneous mixture. Then, water was added gradually at room temperature (varying from 25 to 27 °C – temperature monitored by a thermometer placed in a water bath next to the setup) and stirred for a minimum of 30 min. The water addition was stopped when the mixture became cloudy. The clarity range of some mixtures was extended by placing them into a water bath at 40 °C and allowing them to cool back to room temperature for 5 min. When the mixtures were still transparent, water was added, and the same operation was repeated. When they were not clear anymore, the temperature was maintained at 40 °C while continuing the water addition (Tables S1, S2 and S3).

### Optical properties

The photoconversion experiments were performed by irradiating the NBD solution with a M340L5 LED (340 nm) light source from Thorlabs. The conversion was monitored either by ^1^H-NMR using a 400 or 300 MHz instrument or by UV-Vis using a Jasco V-770 or a Shimadzu 3600 UV-Visible/NIR spectrophotometer (quartz cuvette with a path length of 1 cm).

NBD solutions for the determination of the optical properties were prepared according to the following procedure: NBDs (≈ 2 mg) and Triton™ X-100 reduced (variable weights, detailed per experiment) were first placed into an aluminum weighing boat, which was heated up to 40 °C for 5 min to obtain a clear yellow mixture. The boat was washed with water into a 25 mL volumetric flask, which was then filled with water (*c*_NBD1_ = 3.6 × 10^−4^ M, *c*_NBD2_ = 2.5 × 10^−4^ M). 2748 μL of water were added to 252 μL of the initial solution (*c*_NBD1_ = 3.0 × 10^−5^ M, *c*_NBD2_ = 2.1 × 10^−5^ M).

To evaluate the surfactant content influence on the dissolution of NBDs, the Triton™ X-100 reduced amount added was ranging from 7 to 307 mg for NBD1 and from 6 to 134 mg for NBD2.

The molar extinction coefficients in water were determined by preparing several solutions of NBDs with Triton™ X-100 reduced weights of 118, 202, and 307 mg for NBD1 and 103, 123, and 134 mg for NBD2.

The NBD1 solutions for the kinetics experiments were prepared using the following procedure: an initial solution was prepared (NBD1 (≈ 2 mg, *c*_NBD_ = 3.6 × 10^−4^ M); Triton™ X-100 reduced (≈ 7 mg); water (25 mL)). 1944 μL of water was added to 56 μL of the initial solution (*c*_NBD_ = 1.0 × 10^−5^ M). NBD1 was converted at 340 nm to QC1. A “in-house” setup based on a 3 mL quartz cuvette, an AvaSpec-ULS2048CL-EVO-RS spectrophotometer, and a deuterium Avalight-DHS lamp source from Avantes, coupled with a temperature-controlled sample holder, was used to determine the kinetics parameters.^70^ The spectrum was recorded every 3 min. This was performed for different temperatures, and the rate constants were determined from mono-exponential fits. Thermodynamic parameters (room temperature half-life, activation barrier, enthalpy, and entropy) were obtained from Arrhenius and Eyring plots.

The quantum yield was determined in the same setup, with an irradiation at 340 nm (M340F4 LED from Thorlabs) displaying a photon flux of 1.1044257 × 10 + 14 s^−1^ and using an 80 μL cell (NBD1 (≈ 2 mg), (*c*_NBD_ = 9.0 × 10^−5^ M); Triton™ X-100 reduced (≈ 300 mg); water (= 100 mL)).

To evaluate the catalytic and thermal back-conversions, the QC1 solution in D_2_O/surfactant (NBD1 (≈ 3 mg), (*c*_NBD_ ≈ 0.02 M); Triton™ X-100 reduced (≈ 50 mg); D_2_O (= 500 μL)) was prepared by irradiating a NBD1 solution at 340 nm, in an NMR tube. Then, it was either heated up overnight to 80 °C, or 4.5 mg of CoPc@C were added and left to react for 3 h.

### Integration into a solar harvesting liquid device

The solar simulator used in this work was the ISOSun from infinityPV, calibrated to 1 sun (1000 W m^−2^) with the Sun Calibrated Reference Detector from Sciencetech Inc and the 6430 SUB-FEMTOAMP REMOTE SourceMeter® from Keithley. NBD1 solutions (NBD1 (≈ 312 mg); Triton™ X-100 reduced (≈ 3000 mg); deuterated solvent (= 7300 μL)) were infused using a 10 mL Hamilton 1010 TLL bo STOP syringe and a KD LEGATO 110 I/W Prog Single syringe pump from kdScientific, through a microfluidic chip fabricated from quartz.^9^ The conversion was monitored by a ^1^H-NMR. The inlet and outlet tubing, as well as the collecting vial, were covered with aluminum foil.

### Macroscopic heat release

A solution of NBD1 in toluene was converted under irradiation at 365 nm (16 W), using a Vapourtec flow system (residence time of 30 min, 10 mL loop). The toluene was then evaporated, and 0.6 M solutions were prepared. 400 mg of QC1 was placed in a vial. 2 g of Triton™ X-100 reduced, and 1089 μL of water were added, and the mixture was sonicated to turn clear (solution 1). 2799 μL of toluene was added to 375 mg of QC1 (solution 2). The final solutions were transferred to 10 mL beakers and stirred. 20 mg and 18.8 mg of CoPc@C (5% weight ratio to QC1) were added in one portion to solution 1 and 2, respectively. The heat release was monitored with the Optris PI 640i thermal camera. ^1^H-NMR spectra were recorded before and after to check the conversion and the back-conversion.

Theoretical calculation of the limits of the macroscopic heat release:

The theoretical limits were calculated with eqn (1):1
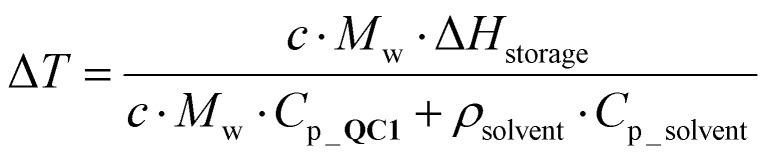
*c* and *M*_w_ represent the concentration of NBD1, 0.6 M, and molecular weight 223.28 g mol^−1^, respectively; Δ*H*_storage_ is the DSC-measured energy storage capacity of the NBD1/QC1 couple, 396 J g^−1^; *C*_p_QC1_ is the specific heat capacity of QC1 in 5.73 J g^−1^ K^−1^, both derived from previous literature^24^ experiments for a concentration of 0.6 M and *ρ*_solvent_ and *C*_p_solvent_ corresponds to the volumetric mass density in g L^−1^ and the specific heat capacity in J g^−1^ K^−1^ of the solvent (there toluene, 867 g L^−1^ and 1.7 J g^−1^ K^−1^, water, 1000 g L^−1^ and 4.18 J g^−1^ K^−1^, Triton™ X-100 reduced, 1029 g L^−1^ and 3.34 J g^−1^ K^−1^, latter experimentally determined).

### Specific heat capacity

The heat capacity of Triton™ X-100 reduced was measured using a Q250 modulated differential scanning calorimeter (MDSC) of TA instruments. Prior to the measurement, the instrument was calibrated using a sapphire standard.

7.78 mg of the sample was sealed in a hermetic aluminium pan. The measurement was carried out under a dry nitrogen atmosphere at a purge flow rate of 50 mL min^−1^. The temperature program ranged from 5 °C to 70 °C with a constant heating rate of 2 °C min^−1^. A sinusoidal modulation of ±1 °C for 120 s was superimposed on the underlying heating rate to allow separation of reversing and non-reversing heat flow components and accurate determination of heat capacity.

Finally, the specific heat value has been taken from the reversing normalized heat capacity data.

### Recovery of NBD1

The converted solution of NBD1 was placed into an oil bath at 80 °C overnight and then allowed to cool down to room temperature. The mixture was transferred into a round-bottom flask and the initial vial was washed with dichloromethane (3 times the vial volume) and distilled water (3 times). A large excess of distilled water was added, and the biphasic system was stirred overnight. An extraction was performed, washing the flask with dichloromethane. The aqueous phase was washed with dichloromethane, and the assembled organic phases were dried over anhydrous sodium sulfate. The oil obtained after filtration and evaporation was purified by column chromatography (9/1 dichloromethane/hexane) (*R*_f_ of NBD1 ≈ 0.7).

## Author contributions

Conceptualization: L. F., Z. W., K. M-P.; formal analysis: L. F.; funding acquisition: K. M-P.; investigation: L. F., H. H., P. F., N. B., K. M.; project administration: H. H., K. M-P.; resources: K. M-P.; supervision: H. H., K. M-P; visualization: L. F.; writing – original draft: L. F.; all authors reviewed the results and approved the final version of the manuscript.

## Conflicts of interest

There are no conflicts to declare.

## Supplementary Material

GC-027-D5GC04357C-s001

GC-027-D5GC04357C-s002

GC-027-D5GC04357C-s003

## Data Availability

The data supporting this article have been included as part of the supplementary information (SI). Supplementary information is available, as well as Supplementary Movies S1 and S2. See DOI: https://doi.org/10.1039/d5gc04357c.
