# Supplementary material for: Surfactant-enabled strategy for molecular solar thermal energy storage systems in water
Source: Green Chem. 2025 Oct 15;27(44):14119–30. doi: 10.1039/d5gc04357c (PMC12539556; doi:10.1039/d5gc04357c)
Supplement: GC-027-D5GC04357C-s001 [file GC-027-D5GC04357C-s001.pdf]

Supplementary Information (SI) for Green Chemistry.
This journal is © The Royal Society of Chemistry 2025

Supporting Information

Surfactant-enabled strategy for molecular solar thermal energy storage systems in water

Lorette Fernandez,[a] Helen Hölzel,*[a,b] Pedro Ferreira,[a] Nicolò Baggi,[a] Kévin Moreno,[a] Zhihang Wang [c,d] and Kasper Moth-Poulsen*[a,e,f,g]

[a] *Department of Chemical Engineering, Universitat Politècnica de Catalunya, EEBE, Eduard Maristany 10–14, 08019 Barcelona, Spain.*
[b] *Institute of Organic Chemistry, Justus-Liebig-University Giessen, Heinrich-Buff-Ring 17, 35392 Giessen, Germany.*
[c] *School of Engineering, College of Science and Engineering, University of Derby, Markeaton Street, Derby DE22 3AW, United Kingdom.*
[d] *Department of Materials Science and Metallurgy, University of Cambridge, Cambridge, CB3 0FS, United Kingdom.*
[e] *Department of Chemistry and Chemical Engineering, Chalmers University of Technology, 41296 Gothenburg, Sweden.*
[f] *The Institute of Materials Science of Barcelona, ICMAB-CSIC, Bellaterra, 08193 Barcelona, Spain.*
[g] *Catalan Institution for Research & Advanced Studies, ICREA, Pg. Lluìs Companys 23, 08010 Barcelona, Spain.*
* *Email: kasper.moth-poulsen@upc.edu*

Contents

1) Surfactants properties 2
2) Ternary diagrams 3
3) Optical properties 6
4) Integration into devices 9
5) Heat release 10
6) NBD1 recovery 12
7) Specific heat capacity of Triton™ X-100 reduced via differential scanning calorimetry 12
References 13

1) Surfactants properties

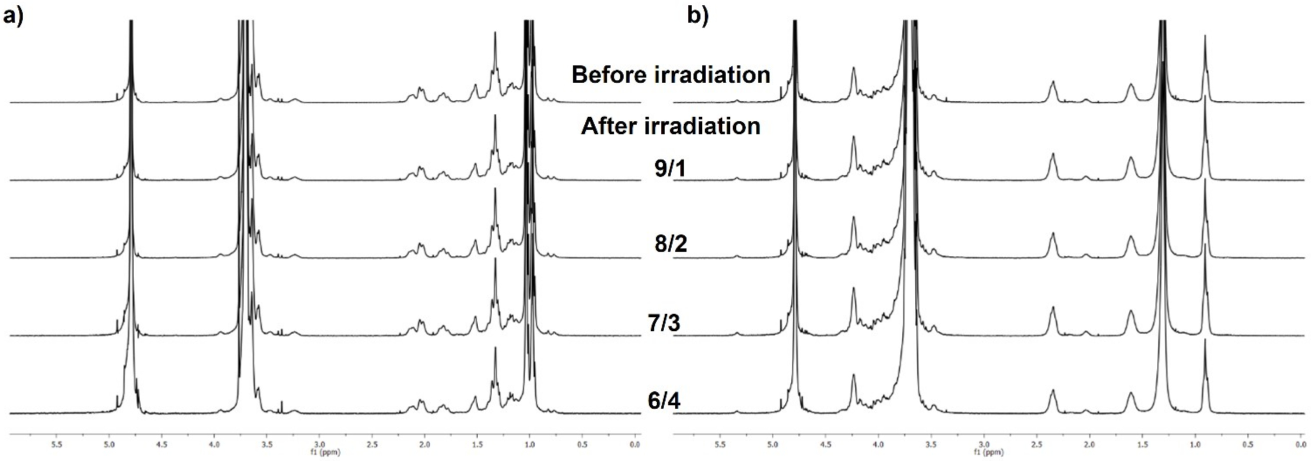

Figure S1. ***Evaluation of the stability of the Triton^{TM} X-100 reduced and TWEEN® 20 under light irradiation at 340 nm for 10 min.*** *a) ^{1}H-NMR (300 MHz, 298 K) spectra of different ratios of Triton^{TM} X-100 reduced in D_2O. b) ^{1}H-NMR (300 MHz, 298 K) spectra of different ratio TWEEN® 20 in D_2O.*

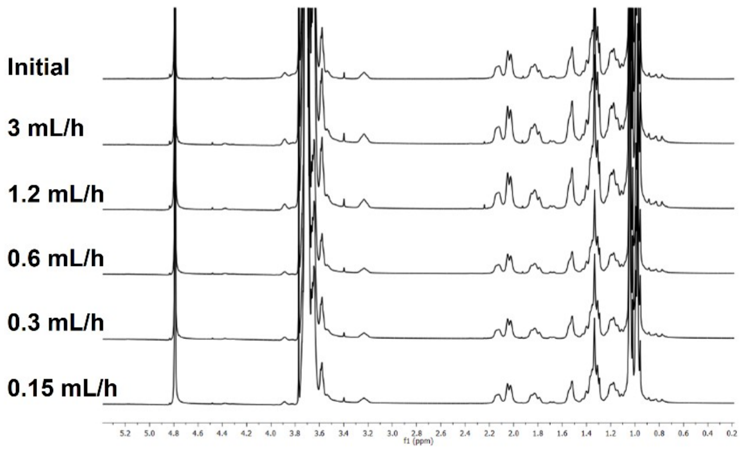

Figure S2. ***Evaluation of the stability of the Triton^{TM} X-100 reduced (28%) in D_2O (72%), under indoors simulated solar irradiation, flowing through a microfluidic chip at different flow rates, monitored by ^{1}H-NMR (400 MHz).***

2) Ternary diagrams

Table S1. Initial ratio of NBD/surfactant (weight percentage). NBD1 weight, Triton^{TM} X-100 reduced (TX-100r) weight, and volume of distilled water used to build the ternary diagram. Weight percentage values from the ternary diagram in Fig. 2 of the manuscript. Final NBD1 concentrations.

Initial wt% NBD1 /surfactant	NBD1 (mg)	TX-100r (mg)	Water (µL)	% NBD1	% TX-100r	% Water	NBD1 (M)
5/95	At r. t.						
	10.2	190	35.4	4	81	15	0.2
			50.2	4	76	20	0.2
			66.9	4	71	25	0.2
			86	4	66	30	0.2
			108	3	62	35	0.2
			133.8	3	57	40	0.1
10/90	At r. t.						
	20.1	179.8	35.4	9	76	15	0.4
			50.2	8	72	20	0.4
			66.9	8	67	25	0.4
			86	7	63	30	0.3
			108	7	58	35	0.3
			133.8	6	54	40	0.3
			164.2	6	49	45	0.3
			200.6	5	45	50	0.2
			245.2	5	40	55	0.2
			301	4	36	60	0.2
			372.6	4	31	65	0.2
			468.1	3	27	70	0.1
15/85	At r. t.						
	29.9	170.1	35.4	13	72	15	0.7
			50.2	12	68	20	0.6
			66.9	11	64	25	0.6
			86	10	60	30	0.5
			108	10	55	35	0.5
	At 40°C						
			133.8	9	51	40	0.4
			164.2	8	47	45	0.4
20/80	At r. t.						
	40.2	159.9	35.4	17	68	15	0.9
			50.2	16	64	20	0.9
			66.9	15	60	25	0.8
	At 40°C						
			86	14	56	30	0.7
			108	13	52	35	0.7
			133.8	12	48	40	0.6

			164.2	11	44	45	0.6
			200.6	10	40	50	0.5
25/75	At 40°C and clear after cooling down to r. t.						
	20.2	59.9	14.2	21	64	15	1.3
			20.1	20	60	20	1.2
	At 40°C						
			26.7	19	56	25	1.1
			34.4	18	52	30	1.0
30/70	At 40°C and clear after cooling down to r. t.						
	15	35.3	8.9	25	60	15	1.6
			12.5	24	56	20	1.4

Table S2. Initial ratio of NBD/surfactant (weight percentage). NBD1 weight, TWEEN® 20 (Tw20) weight, and volume of distilled water used to build the ternary diagram. Weight percentage values from the ternary diagram in Fig. 2 of the manuscript. Final NBD1 concentrations.

Initial wt% NBD1 /surfactant	NBD1 (mg)	Tw20 (mg)	Water (µL)	% NBD1	% Tw20	% Water	NBD1 (M)
5/95	At r. t.						
	9.8	190	35.4	4	81	15	0.2
			50.2	4	76	20	0.2
			66.9	4	71	25	0.2
			86	3	67	30	0.2
			108	3	62	35	0.2
			133.8	3	57	40	0.1
10/90	At r. t.						
	20	179.9	35.4	9	76	15	0.4
			50.2	8	72	20	0.4
			66.9	8	67	25	0.4
			86	7	63	30	0.4
15/85	At r. t.						
	29.8	169.8	35.4	13	72	15	0.7
			50.2	12	68	20	0.7

Table S3. Initial ratio of NBD/surfactant (weight percentage). NBD2 weight, Triton™ X-100 reduced (TX-100r) weight, and volume of distilled water used to build the ternary diagram. Weight percentage values from the ternary diagram in Fig. 2 of the manuscript. Final NBD2 concentrations.

Initial wt% NBD2 /surfactant	NBD2 (mg)	TX-100r (mg)	Water (µL)	% NBD2	% TX-100r	% Water	NBD2 (M)
5/95	At r. t.						
	9.9	192.8	35.4	4	81	15	0.2
			50.2	4	76	20	0.2
			66.9	4	72	25	0.2
			86	3	67	30	0.2
			108	3	62	35	0.2
			133.8	3	57	40	0.1
10/90	At r. t.						
	20.1	180.4	35.4	9	77	15	0.4
			50.2	8	72	20	0.4
			66.9	8	68	25	0.4
14/86	At r. t.						
	30.2	182.1	35.4	12	74	14	0.6
			50.2	12	69	19	0.6

3) Optical properties

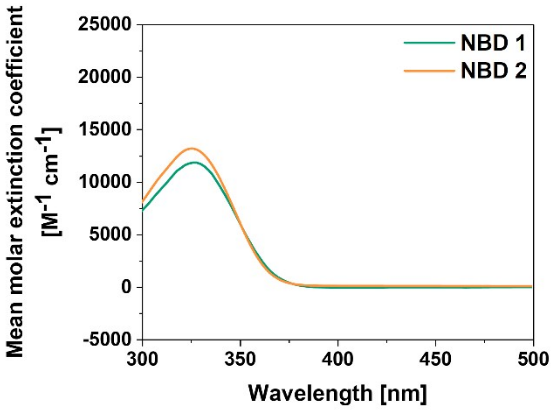

Figure S3. ***Calculated mean molar extinction coefficient values, determined by UV-vis spectroscopy.***

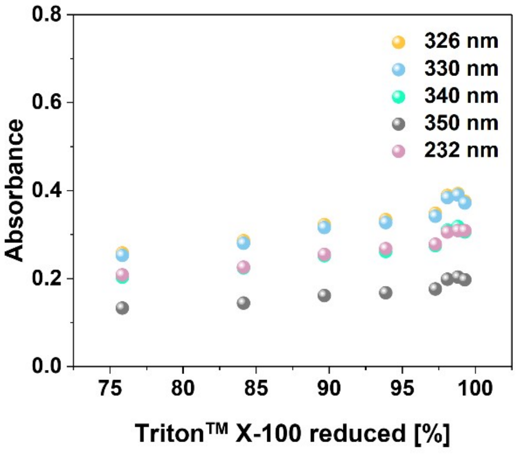

Figure S4. ***Dissolution evaluation. Absorbance of NBD1 at different wavelengths, function of the Triton™ X-100 reduced weight, monitored by UV-vis spectroscopy.***

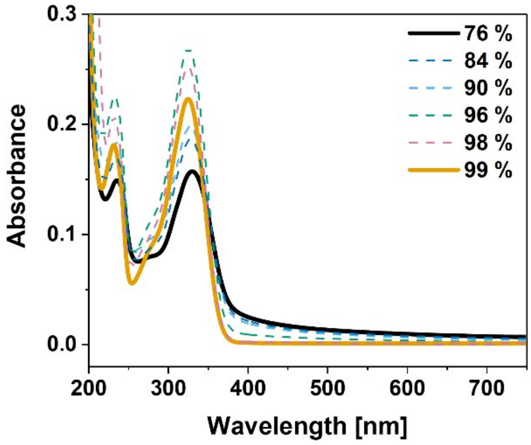

Figure S5. ***Dissolution/aggregation evaluation. Evolution of the absorbance spectra of NBD2 function of the Triton^{TM} X-100 reduced weight percentages, monitored by UV-vis spectroscopy.***

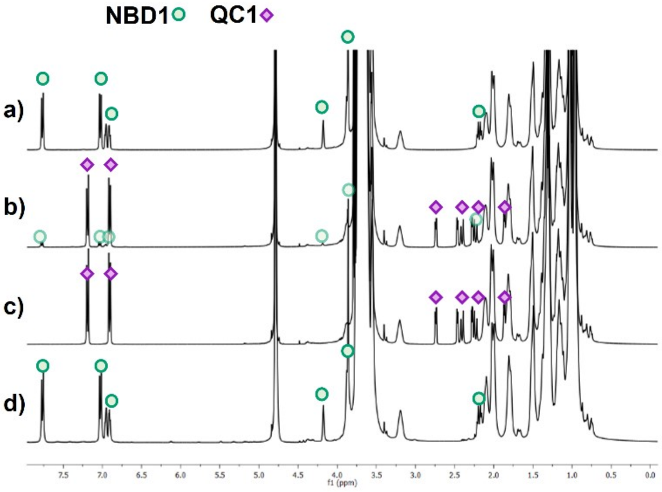

Figure S6. ***Conversion and back-conversion of NBD1 (0.02 M) in D_2O and Triton^{TM} X-100 reduced monitored by ^{1}H-NMR (400 MHz).*** *a) Initial spectrum. b) Spectrum after irradiation at 340 nm for 2 h 20 min. c) Spectrum after irradiation at 340 nm for 3 h 25 min. d) Spectrum after heating up the sample at 80°C overnight.*

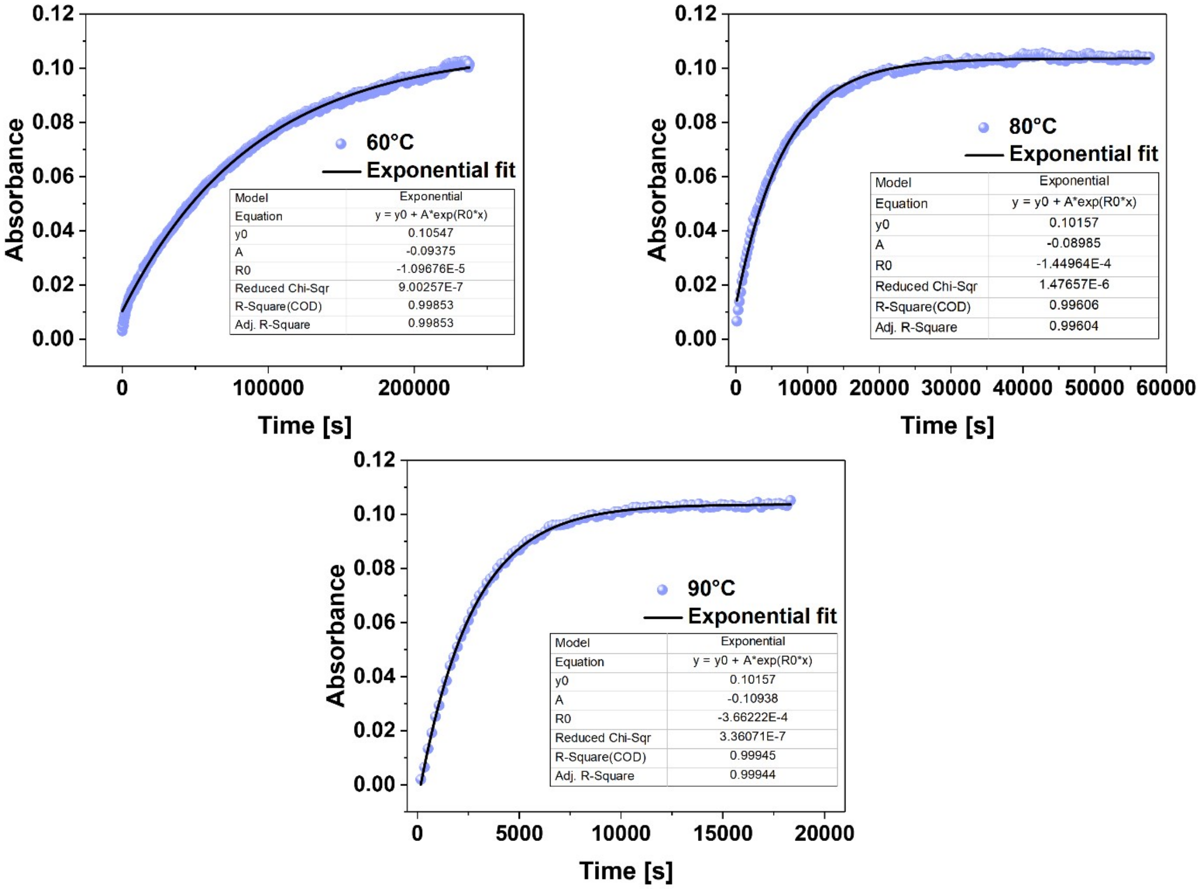

Figure S7. ***Kinetics plots of QC1 to NBD1 back-conversion at different temperatures in a water-based solution.***

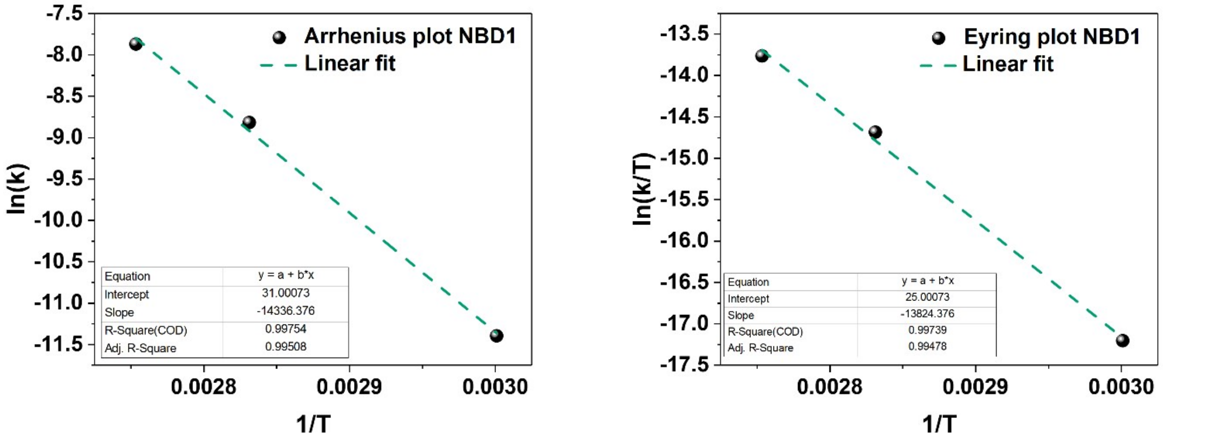

Figure S8. ***Arrhenius and Eyring plots from the back-conversion rates of QC1 to NBD1 at different temperatures in a water-based solution.***

Table S4. Determination of the quantum yield of NBD1 in a water-based solution.

Sample	QY (%)
1	81
2	79
3	79
4	78
MEAN	**79 ± 1**

4) Integration into devices

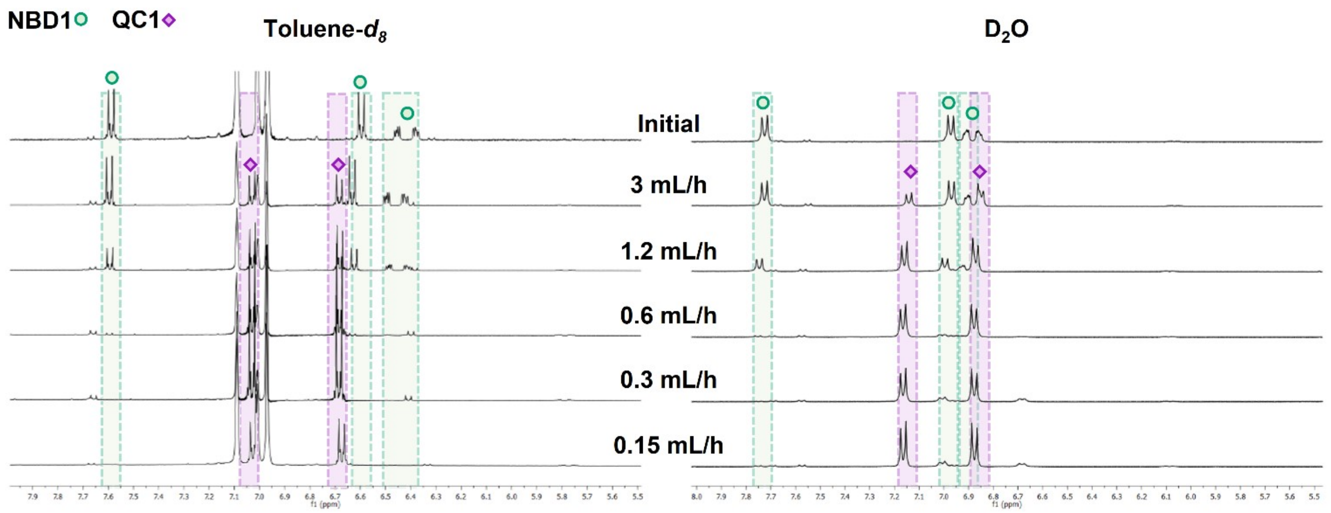

Figure S9. ***Conversion under indoor simulated solar irradiation of solutions of NBD1 in toluene-d8 and in D_2O with Triton^{TM} X-100 reduced, flowing through a microfluidic chip at different flow rates, monitored by ^1H-NMR (400 MHz).***

Table S5. Flow rates, conversion percentages, and energy storage efficiencies generated over different residence times for NBD1 during the indoor experiment testing. Values used in the graph in Figure X of the manuscript.

In toluene

Flow rate (mL.h^{-1})	Residence time (s)	Conversion (%)
3	40.7	37.6
1.2	101.7	64.1
0.6	203.4	96.2
0.3	406.8	100
0.15	813.6	100

In water

Flow rate (mL.h^{-1})	Residence time (s)	Conversion (%)
3	40.7	36.9
1.2	101.7	65.4
0.6	203.4	98.0
0.3	406.8	99.0
0.15	813.6	97.1

5) Heat release

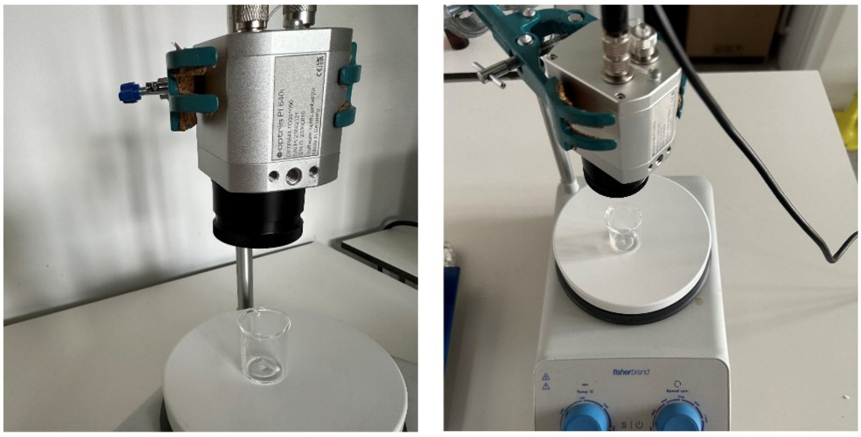

Figure S10. ***Pictures of the heat release set up.***

a)

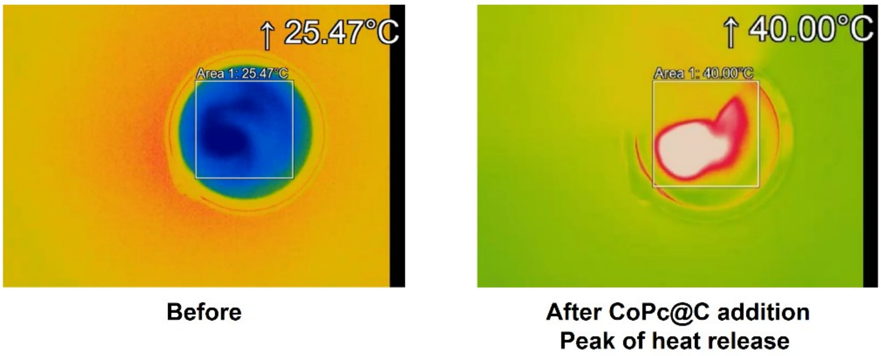

b)

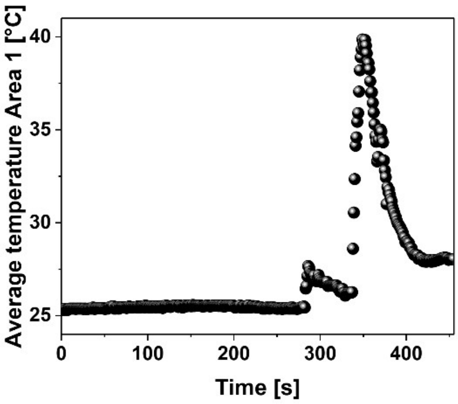

Figure S11. ***Reference experiment of heat release in toluene. a) Thermal images before and after the addition of the catalyst to the QC1 solution (0.6 M). b) Evolution of the average temperature of Area 1.***

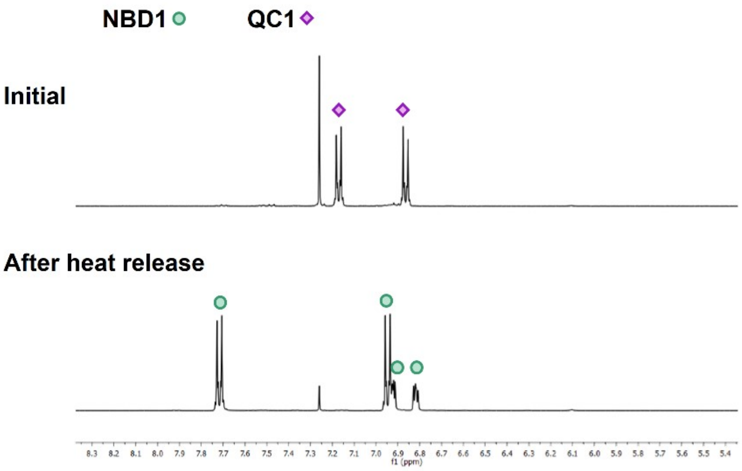

Figure S12. ***Catalytic back-conversion in toluene during the heat release experiment, monitored by*** ^{1}***H-NMR (400 MHz), in*** $CDCl_3$.

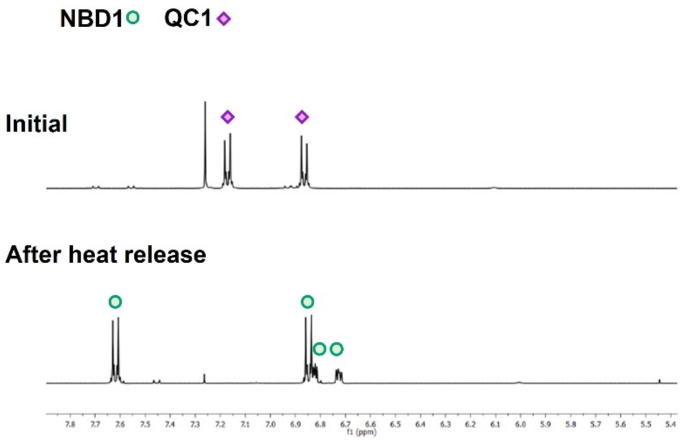

Figure S13. ***Catalytic back-conversion in TX100r/water during the heat release experiment, monitored by*** ^{1}***H-NMR (400 MHz), in*** $CDCl_3$.

6) NBD1 recovery

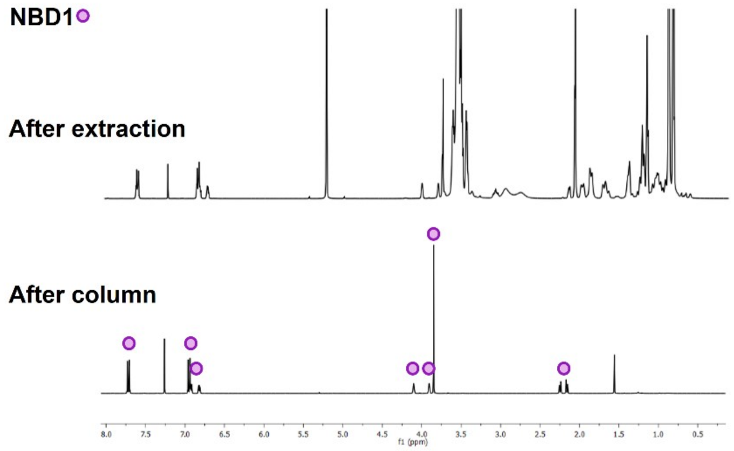

Figure S14. ***Recovery of NBD1 monitored by*** ^{1}***H-NMR (400 MHz), in*** $CDCl_3$***. Extraction with dichloromethane followed by column chromatography.***

7) Specific heat capacity of Triton™ X-100 reduced via differential scanning calorimetry

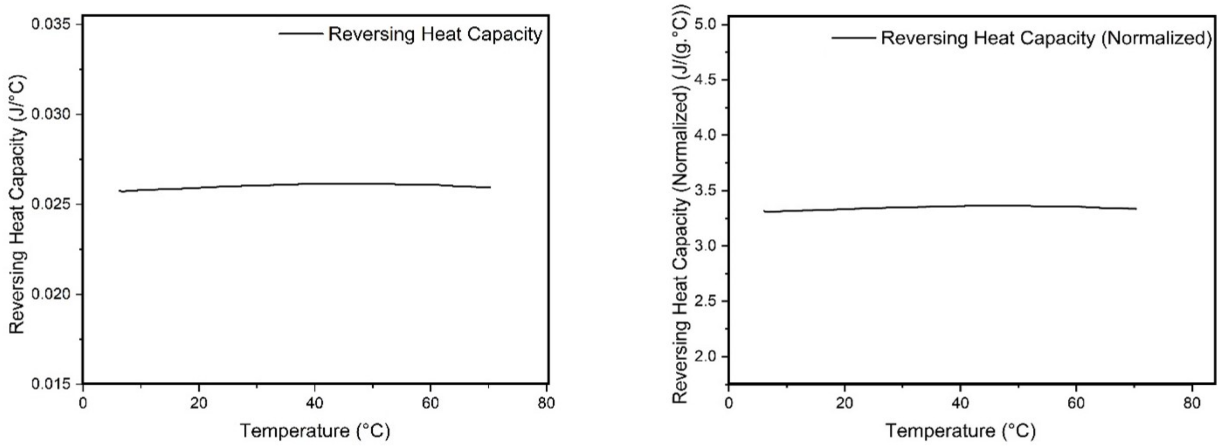

Figure S15. Left: experimentally determined heat capacity via differential scanning calorimetry and right: specific heat capacity.

References

(1) Wang, Z.; Roffey, A.; Losantos, R.; Lennartson, A.; Jevric, M.; Petersen, A. U.; Quant, M.; Dreos, A.; Wen, X.; Sampedro, D.; Börjesson, K.; Moth-Poulsen, K. Macroscopic Heat Release in a Molecular Solar Thermal Energy Storage System. *Energy Environ. Sci.* **2019**, *12* (1), 187–193. https://doi.org/10.1039/C8EE01011K.

(2) Wang, Z.; Wu, Z.; Hu, Z.; Orrego-Hernández, J.; Mu, E.; Zhang, Z.-Y.; Jevric, M.; Liu, Y.; Fu, X.; Wang, F.; Li, T.; Moth-Poulsen, K. Chip-Scale Solar Thermal Electrical Power Generation. *Cell Reports Physical Science* **2022**, *3* (3), 100789. https://doi.org/10.1016/j.xcrp.2022.100789.
